# The Dynamic of PRAMEY Isoforms in Testis and Epididymis Suggests Their Involvement in Spermatozoa Maturation

**DOI:** 10.3389/fgene.2022.846345

**Published:** 2022-03-21

**Authors:** Chandlar H. Kern, Weber B. Feitosa, Wan-Sheng Liu

**Affiliations:** Department of Animal Science, Center for Reproductive Biology and Health, College of Agricultural Sciences, The Pennsylvania State University, University Park, PA, United States

**Keywords:** PRAMEY, cancer/testis antigen, spermatogenesis, sperm maturation, cattle

## Abstract

The preferentially expressed antigen in melanoma, Y-linked (PRAMEY) is a cancer/testis antigen expressed predominantly in bovine spermatogenic cells, playing an important role in germ cell formation. To better understand PRAMEY’s function during spermatogenesis, we studied the dynamics of PRAMEY isoforms by Western blotting (WB) with PRAMEY-specific antibodies. The PRAMEY protein was assessed in the bovine testicular and epididymal spermatozoa, fluid and tissues, and as well as in ejaculated semen. The protein was further examined, at a subcellular level in sperm head and tail, as well as in the subcellular components, including the cytosol, nucleus, membrane, and mitochondria. RNA expression of PRAMEY was also evaluated in testis and epididymal tissues. Our WB results confirmed the previously reported four isoforms of PRAMEY (58, 30, 26, and 13 kDa) in the bovine testis and spermatozoa. We found that testicular spermatozoa expressed the 58 and 30 kDa isoforms. As spermatozoa migrated to the epididymis, they expressed two additional isoforms, 26 and 13 kDa. Similarly, the 58 and 30 kDa isoforms were detected only in the testis fluid, while all four isoforms were detected in fluid from the cauda epididymis. Tissue evaluation indicated a significantly higher expression of the 58 and 13 kDa isoforms in the cauda tissue when compared to both the testis and caput tissue (*p* < 0.05). These results indicated that testis samples (spermatozoa, fluid, and tissue) expressed predominantly the 58 and 30 kDa PRAMEY isoforms, suggesting their involvement in spermatogenesis. In contrast, the 26 kDa isoform was specific to epididymal sperm and the 13 kDa isoform was marked in samples derived from the cauda epididymis, suggesting their involvement in sperm maturation. Results from the sperm head and tail experiments indicated that the 13 kDa isoform increased 4-fold in sperm tails from caput to cauda, suggesting this isoform may have a significant role in tail function. Additionally, the 13 kDa isoform increased significantly (*p* < 0.05) in the cytosol during epididymal passage and tended to increase in other subcellular components. The expression of PRAMEY in the sperm subcellular components during epididymal maturation suggests the involvement of PRAMEY, especially the 13 kDa isoform, in sperm motility.

## Introduction

Spermatozoa are formed throughout a male’s reproductive lifetime from spermatogonial stem cells (SSCs) and function as the male reproductive cells that contribute to fertilization. Spermatogenesis takes place in the seminiferous tubules of the testis through three phases: mitosis, meiosis, and spermiogenesis. The last phase, spermiogenesis, represents the process where haploid round spermatids are converted into fully differentiated spermatozoa and are released into the lumen of the seminiferous tubules (Hess and de Franca, 2008).

During spermatogenesis, numerous germ cell-specific antigens are expressed, however many of these antigens are nonexistent or are minimally detected in normal somatic tissues, while they are highly expressed in various tumors (Chang et al., 2011). Because these antigens have restricted expression in the testis and cancer cells, they have been termed cancer/testis antigens (CTAs). During spermatogenesis, numerous CTAs are detected at a specific stage (e.g., synaptonemal complex protein 1, SCP1) (Meuwissen et al., 1992), while others are found at multiple time points during spermatogenesis (e.g. trophinin, TRO, preferentially expressed antigen in melanoma-like 1, PRAMEL1, and Prame family 12, PRAMEF12) (Saburi et al., 2001; Mistry et al., 2013; Wang et al., 2019; Kern et al., 2021). The stage-specific appearance of CTAs throughout the spermatogenic process has led researchers to believe that there is significant importance associated with CTA emergence and their function in spermatogenesis (Tulsiani et al., 1998).

One of the CTAs, the PRAME protein, was originally discovered in a human melanoma cell line (Ikeda et al., 1997). Primary research in cancer biology found PRAME to be a dominant repressor of the retinoic acid receptor (RAR) in melanoma cells (Epping et al., 2005, 2008). Later studies indicated that PRAME was involved in nuclear factor Y (NFY)-mediated transcriptional regulation as a subunit of a Cullin2-based E3 ubiquitin ligase in leukemia cells (Costessi et al., 2011). Members of the *PRAME* gene family encode leucine-rich repeat (LRR) proteins that fold into a horseshoe shape, which provides a versatile structural framework for the formation of protein-protein interactions (Kobe and Kajava, 2001; Epping et al., 2005; Wadelin et al., 2010). Protein phosphatase 1, regulatory (inhibitor) subunit 7 (PPP1R7), also known as SDS22, is another LRR protein and interacts with protein phosphatase 1 catalytic subunit gamma isozyme (PPP1CC), also known as PP1γ2, in bovine cauda epididymal spermatozoa (Huang et al., 2002; Mishra et al., 2003). As a testis/spermatozoa-specific phosphatase, PP1γ2 acts as an important regulator of sperm motility, and male fertility (Smith et al., 1996; Fardilha et al., 2011). While the regulatory function of PRAME in cancer cells is well studied (Epping et al., 2005; Costessi et al., 2011), the role of the PRAME protein family in germ cells, and male reproduction is still unclear.

Through evolution, the *PRAME* gene family has been amplified and it constitutes a large gene family in eutherian mammals (Birtle et al., 2005; Church et al., 2009; Chang et al., 2011). In bovine, *PRAME* consists of multiple copies in chromosome 16, with a single copy in chromosome 17. An autosome (i.e., chromosome 17)-to-Y transposition and subsequent amplification resulted in a Y-linked *PRAME* gene (*PRAMEY*) sub-family (Chang et al., 2011, 2013). This transposition event occurred during bovine evolution and is believed to enhance bovid male fertility (Chang et al., 2011). Additionally, the copy number variation (CNV) of PRAMEY has been found to correlate with male reproductive traits and could possibly be a valuable marker for male fertility selection (Yue et al., 2013).

Previous studies identified four PRAMEY isoforms (58, 30, 26, and 13 kDa) within the bovine testes tissue (58 and 30 kDa isoforms) and/or epididymal spermatozoa (30, 26, and 13 kDa isoforms) using a custom-made PRAMEY-specific antibody (Liu et al., 2017). The bovine PRAMEY was also found to be enriched in the intermitochondrial cement (IMC) and chromatoid body (CB) of bovine spermatogenic cells (Liu et al., 2017). IMC and CB represent a class of mammalian spermatogenic cell-specific organelles known as germinal granules and commonly referred to as nuage (Meikar et al., 2011). The IMC originates in the cytoplasm of spermatocytes, but it can no longer be located after meiosis. However, the CB is present in the cytoplasm of post-meiotic spermatids. Therefore, PRAMEY’s presence in these organelles signifies its importance in spermatogenesis.

Given the knowledge of PRAMEY’s possible role in spermatogenesis, the objective of this study was to characterize the detailed expression pattern of the PRAMEY protein within spermatozoa, reproductive fluids and tissues of the bovine testis, epididymis, and ejaculated semen. Ultimately, our goal was to use the expression and localization data gained from this study to understand the functional role of PRAMEY during sperm formation, maturation, and function.

## Material and Methods

### Sample Collection

Pairs of mature bovine testes with intact epididymides were obtained from a local slaughter house (Nicholas Meat, LLC, Loganton, PA, United States). Testes were transported to the laboratory in ice-filled coolers and processed within 2 h to collect spermatozoa, fluid, and tissues from the testes and epididymides (see following sections for specific collection procedures).

For the experiments that evaluated PRAMEY expression in spermatozoa, fluid, and tissue, samples were collected from five pairs of mature testes with intact epididymides (*n* = 5). For the experiments that evaluated the PRAMEY expression in sperm head and tail, as well in the sperm subcellular fraction, spermatozoa were collected from three pairs of mature testes with intact epididymides (*n* = 3). In the experiments where mRNA expression was analyzed, the samples (testis, caput, corpus, and cauda epididymis) were collected from three animals (*n* = 3). For the experiments using freshly ejaculated semen and seminal plasma, the samples were collected from nine yearling beef bulls (*n* = 9) from the Pennsylvania Department of Agriculture Livestock Evaluation Center.

### Testicular and Epididymal Sperm Collection

Epididymides were removed from the testis and dissected into caput and cauda segments. The PRAMEY mRNA expression experiment was the only one to include the corpus epididymal segment. Sperm collection procedures were adapted from protocols previously established in our laboratory (Liu et al., 2017). Briefly, the isolated testicles were washed with phosphate-buffered saline (PBS; 137 mM NaCl, 2.7 mM KCl, 10 mM Na2PO4, and 1.8 mM KH2PO4, pH 7.4) and opened by a sagittal cut using a scalpel. By applying light pressure to the testis, the testicular fluid containing spermatozoa was collected, transferred to a 2 ml Eppendorf tube, and centrifuged at 1,500 x g for 10 min at 4 °C to remove the testicular spermatozoa (the supernatant was reserved). The testicular sperm pellet was washed twice in 2 ml PBS at 1,500 x g for 10 min at 4°C and the resulting sperm pellet was stored at −80°C. The dissected caput or cauda epididymides were washed in PBS and placed in a Petri dish. The epididymal tissue was cut with a scalpel and the fluid containing the spermatozoa was collected by applying pressure on the caput or cauda epididymides. The epididymal fluid was transferred to a 2 ml Eppendorf and centrifuged at 1,500 x g for 10 min at 4°C to collect the epididymal spermatozoa (the supernatant was reserved). The caput and cauda sperm pellets were washed twice in 2 ml PBS at 1,500 x g for 10 min at 4°C and the resulting sperm pellets were stored at −80°C. For the sperm head and tail separation portion of the project*,* the caput or cauda epididymal spermatozoa were isolated by slicing the epididymal tissue in a Petri dish with PBS. The isolated spermatozoa were centrifuged at 1,500 x g for 10 min at 4°C, and the caput and cauda sperm pellets were used to fractionate the spermatozoa into head and tail portions by sonication.

### Testis and Epididymal Fluid Collection

After collecting spermatozoa from the bovine testis and epididymis as described above, the reserved supernatants were transferred to new 2 ml Eppendorf tubes and centrifuged at 4,000 x g for 20 min at 4°C to remove cellular debris. The testis, caput and cauda fluids were stored at −80°C.

### Testis and Epididymal Tissue Collection

Tissue collection procedures were adapted from protocols previously established in our laboratory (Liu et al., 2017). Tissues from the testis and caput and cauda segments of the epididymis were collected using a scalpel, cut into small pieces, and washed twice in PBS. A portion of the tissues were snap frozen in liquid nitrogen and stored at −80°C until further use for protein and RNA extraction.

### Ejaculated Semen Collection

Ejaculated semen was obtained from yearling beef bulls at the Pennsylvania Department of Agriculture Livestock Evaluation Center from animals within the Bull Testing Program. The bulls were assessed through breeding soundness evaluation and electroejaculation was used to obtain semen by a certified veterinarian. All animal procedures were performed in accordance with the Guide for the Care and Use of Laboratory Animals and approved by the Animal Care and Use Committees of the Pennsylvania State University. The collected semen was donated for our research. Semen was collected in 15 ml tubes, kept on ice for the duration of collection, and was transported to the laboratory in ice-filled coolers.

### Sperm Head and Tail Separation

The sperm head and tail separation procedure was adapted from Tateno and co-workers (Tateno et al., 2000). The isolated spermatozoa from epididymal fluid were washed three times in PBS (containing 137 mM NaCl, 2.7 mM KCl, 10 mM Na2PO4, and 1.8 mM KH2PO4, pH 7.4) with centrifugation at 1,100 x g for 3 min at 4°C. Each sample was sonicated for 30 s at 8 W of power (Fisher Scientific, Sonic Dismembrator Model 100) to break the sperm heads from the tails. To separate the head and tail fractions of the sonicated mixture, 500 uL of Tris Buffer Saline (TBS) (containing 50 mM Tris-Cl, 150 mM NaCl, pH 7.6) was added to each sperm sample and then mixed by vortexing. The diluted spermatozoa (200 uL) were added to 1.0 ml of a 90% Percoll solution (GE Healthcare, product no. 17-0891-02) and the samples were centrifuged at 15,000 x g for 15 min at room temperature (RT). After centrifugation, the sperm tails were located in a thin band near the top of the 1.5 ml tube, and the sperm heads were located in a pellet at the bottom of the tube. The sperm heads and tails were removed separately from the 90% Percoll gradient and put into new 1.5 ml tubes to wash. The sperm heads and tails were washed in 500 uL of TBS (containing 50 mM Tris-Cl, 150 mM NaCl, pH 7.6) at 9,000 x g for 5 min at RT and were immediately frozen in liquid nitrogen.

### Subcellular Fractionation

Subcellular fractionation was performed following the Abcam subcellular fractionation protocol (https://www.abcam.com/protocols/subcellular-fractionation-protocol). Briefly, sperm cells from caput and cauda epididymides were resuspended in 500 μl fractionation buffer and (20 mM HEPES, 10 mM KCL, 2 mM MgCl2, 1 mM EDTA, and 1 MM EGTA supplemented with 1 mM DTT), protease and phosphatase inhibitor cocktails. They were homogenized and incubated for 20 min on ice and were then centrifuged at 720 x g for 5 min. The supernatant containing the sperm cytoplasm, plasma membrane, and mitochondria was transferred into a fresh tube and kept on ice, while the pellet containing nuclei was washed with 500 μl fractionation buffer. The nuclear pellet was dispersed by vortexing, and it was then centrifuged again at 720 x g for 10 min. The resulting pellet was resuspended in TBS with 0.1% SDS, sonicated briefly to shear genomic DNA and homogenize the lysate (3 s on ice at a power setting of 2-continuous), and stored at −80°C. The supernatant containing the sperm cytoplasm, membrane, and mitochondria was centrifuged at 10,000 x g for 5 min at 4°C. The supernatant containing the cytoplasm and membrane was transferred into a fresh tube and kept on ice, while the pellet containing the mitochondria was processed as described for the nuclear pellet to obtain mitochondrial lysate in TBS/0.1% SDS. The supernatant containing the cytoplasm and membrane was centrifuged at 50,000 x g for 2 h. The supernatant containing the cytosol was stored at −80°C, while the pellet was resuspended in 400 μl of fractionation buffer supplemented with 1% Triton and re-centrifuged for 45 min under the same conditions. The resulting membrane pellet was processed as described for the nuclear pellet.

### Protein Extraction

Protein extraction procedures were adapted from protocols previously established in our laboratory (Liu et al., 2017). Protein was extracted from testicular and epididymal spermatozoa, fluids, and tissues, and as well as from sperm heads and tails for this project. Protein was extracted using CelLytic buffer (Sigma, product no. C3228) added with protease inhibitor cocktail (Thermo Scientific, product no. 1860932) and phosphatase inhibitor cocktail (Thermo Scientific, product no. 1862495). The testes and epididymal sperm, fluid, and tissues, and sperm head and tail pellets were removed from the −80°C freezer and ice-cold extraction buffer (supplemented with protease inhibitor and phosphatase inhibitor cocktails) was immediately added to the pellets. The mixture was homogenized on ice and then incubated on ice for 10 min at 4°C on a shaker. The samples were centrifuged for 10 min at 13,200 x g at 4°C. The supernatants were removed and were referred to as the respective protein for each testis and epididymal sperm, fluid, tissue, and sperm head, and tail samples in this study.

### RNA Extraction, cDNA Synthesis, and RT-PCR

The following kits were used for RNA extraction from the testis, and the caput, corpus, and cauda epididymal tissues: The miRNAeasy Mini Kit (Qiagen cat no. 217004), QIAshredder spin columns (Qiagen cat no. 79654), and the RNase-free DNase set (Qiagen cat no. 79254). The protocol for RNA extraction was performed following the “Purification of Total RNA, Including Small RNAs, from Animal Tissues” described in the miRNeasy Mini Handbook (file:///C:/Users/wul12/AppData/Local/Temp/HB-1253003_HB_miRNeasy_96_1,120_%20WW.pdf). cDNA was synthesized using the Superscript III First-Strand Synthesis System for reverse transcription (RT)-PCR (Invitrogen product no. 18080051). The cDNA synthesis was performed following the protocol under “First-strand cDNA synthesis” within the product handbook. RT-PCR was performed using the cDNA samples, The PCR protocol was as follows: each 20-μl reaction contained 13.76 μl of distilled water, 0.5 μl of each primer (10 pmol/μl), 4 μL of Bioline 5× buffer (Bioline United States Inc., Taunton, MA, including 200 μM deoxyribonucleotide triphosphates), 0.24 μl of Bioline Taq DNA polymerase (Bioline United States Inc.), and 1 μl of either genomic DNA (10 ng/μl) or water. Thermocycling for the gene of interest, PRAMEY (forward, 5′- TCA​GGA​CCT​GGA​GGT​CAA​C-3’; reverse, 5′-TGT​GGC​AAT​ATG​TGG​ATG​CG-3′), consisted of an initial denaturation at 95°C for 5 min, followed by 35 cycles of at 94°C for 30 s, at 65°C for 30 s, and 72°C for 30 s, and a final extension at 72°C for 5 min. Thermocycling for the control gene, GADPH (forward, 5′- AAC​GGA​TTT​GGC​CGT​ATT​GG-3’; reverse, 5′-CAT​TCT​CGG​CCT​TGA​CTG​TG-3′), consisted of an initial denaturation at 95°C for 5 min, followed by 35 cycles of at 94°C for 30 s, at 55°C for 30 s, and 72°C for 30 s, and a final extension at 72°C for 5 min.

### Gel Electrophoresis and Western Blotting

Gel electrophoresis and WB procedures were adapted from protocols previously established in our laboratory (Liu et al., 2017). The protein extracts were separated by a 4–12% acrylamide gel. The gels were electronically transferred to polyvinylidene difluoride (PVDF) membranes (Millipore, product no. IPVH00010). The membranes were blocked in 5% nonfat dried milk (NFDM) in tris buffered saline containing 0.05% Tween-20 (TBST). After being briefly washed in TBST, the membrane was incubated in the primary antibody, a PRAMEY-specific (Liu et al., 2017) or PRAMEYc custom antibody produced by New England Peptide, LLC (Gardner, MA, United States) at a dilution of 1:500, overnight at 4°C. The membranes were washed with TBST 3 times for 10 min each and then incubated in the secondary antibody, anti-rabbit IgG, HRP linked (Cell Signaling Technology, product no. 7074S, 1:5,000 dilution) for 1 h at room temperature. The membranes were washed 3 times for 10 min each and the reactive proteins were detected by SuperSignal West Femto Maximum Sensitivity Substrate (Thermo Scientific, product no. 34095). After PRAMEY or PRAMEYc antibody incubation, membranes were stripped with 15 ml of stripping buffer (10% SDS, 0.5 M Tris HCl, β-mercaptoethanol) for 30 min at 50°C. Membranes were washed with TBST 6 times for 5 min each and then re-blocked in 5% NFDM. Alpha tubulin (TUBA) primary antibody (Cell Signaling Technology, product no. 3873, 1:1,000 dilution) was used to probe the membranes again overnight at 4°C. The membranes were washed with TBST 3 times for 10 min each and then incubated in the secondary antibody, anti-mouse IgG, HRP linked (Cell Signaling Technology, product no. 7076, 1:5,000 dilution) for 1 h at room temperature. The membranes were washed 3 times for 10 min each and the reactive proteins were detected by SuperSignal West Femto Maximum Sensitivity Substrate (Thermo Scientific, product no. 34095).

### Statistical Analysis

WB images were analyzed using ImageJ software (Schneider et al., 2012). The data were analyzed by the normality test (Shapiro-Wilk test) and equal variance test (Brown-Forsythe) using SigmaPlot 12.0 (Statistical Software). After meeting the assumptions of normally distributed data and homogeneity of variance, the difference in treatment levels was compared by one-way ANOVA with the post-hoc *Tukey test*. Data are expressed as the mean ± SEM, and a value of *p* < 0.05 was considered statistically significant.

## Results

### The Pattern of PRAMEY Expression in Bovine Sperm

**FIGURE 1:**
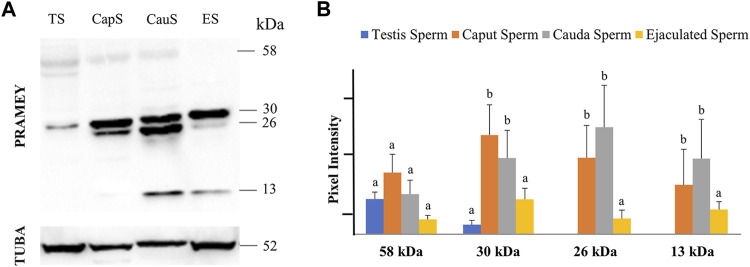
Expression of PRAMEY isoforms in bovine spermatozoa collected from testis (TS), caput (CapS), and cauda (CauS) segments of the epididymis and ejaculated spermatozoa (ES). **(A)** Immunoblot of total sperm protein in each group probed by PRAMEY antibody and then reprobed by α-tubulin (TUBA) antibody. **(B)** Quantification of pixel intensities for the 58, 30, 26, and 13 kDa PRAMEY bands, normalized to the corresponding TUBA. Data are expressed as the mean of the protein pixel intensity ±SEM (*n* = 5). Significance was evaluated between the four types of sperm (testis, caput, cauda, ejaculated) for each PRAMEY isoform (58, 30, 26, and 13 kDa). Values without a common superscript differed (*p* < 0.05).

In a previous report, four different isoforms of the bovine PRAMEY protein including the 58, 30, 26, and 13 kDa isoforms were identified by WB with a PRAMEY-specific antibody (Liu et al., 2017). In the current study, all four isoforms of PRAMEY were found to be present in testicular, epididymal, and ejaculated spermatozoa of mature adult bulls (Figure 1A). The 58 kDa protein, which is the predicted molecular weight for the PRAMEY was detected, but at a low amount in spermatozoa (Table 1) from testis, caput, and cauda of the epididymis and ejaculated sperm (*p* > 0.05) (Figure 1A,B). The 30 kDa isoform had a higher expression in epididymal spermatozoa (Table 1) compared to testicular and ejaculated sperm (*p* < 0.05) (Figure 1A,B). As spermatozoa moved to the epididymis, two additional isoforms, 26 and 13 kDa were detected. Although there was no difference (*p* > 0.05) in the 26 kDa protein amount between caput and cauda sperm, a significant difference was found between epididymal and ejaculated sperm (*P˂*0.05). The protein level of the 13 kDa isoform was similar (*p* > 0.05) between cauda and caput sperm. Comparable to the 26 kDa isoform, the 13 kDa protein amount was higher in epididymal sperm compared to ejaculated sperm (Figure 1A,B). These results suggest that the two smaller isoforms of PRAMEY may be involved in sperm maturation and sperm fertilizing ability. However, it is unknown whether the 26 and 13 kDa proteins are new isoforms or they are products of the 58 and 30 kDa isoform cleavage/degradation.

**TABLE 1:** Summary of PRAMEY isoforms detected in bovine sperm, fluid, and tissue by WB^a^.

PRAMEY	58 kDa	30 kDa	26 kDa	13 kDa
Testis sperm	+	+	−	−
Caput sperm	+	+ + + +	+ + +	+ +
Cauda sperm	+	+ + +	+ + + +	+ + +
Ejaculated sperm	+	+ +	+	+
Testis fluid	+	+ +	−	−
Caput fluid	+	+ + +	-	+
Cauda fluid	+	+ +	+	+ +
Seminal Plasma	+	+	−	+ +
Testis tissue	+	+ + +	−	−
Caput tissue	+	+ + +	−	+
Cauda tissue	+	+ + +	−	+ +

a Note: +/−: PRAMEY, isoforms were either detected (+) or not detected (−) by the PRAMEY, antibody; +: low detection; ++: medium detection; +++/++++: high detection.

### Distribution of PRAMEY in Testicular and Epididymal Fluid and Seminal Plasma

**FIGURE 2:**
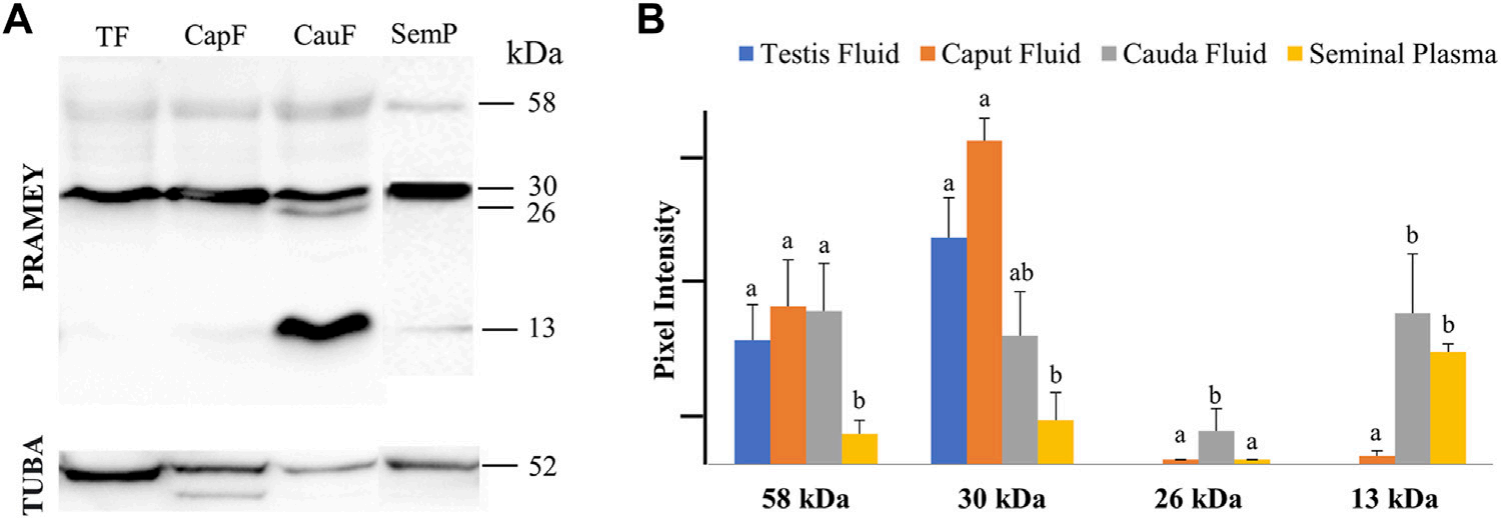
Expression of PRAMEY isoforms in bovine testis (TF), caput epididymal fluid (CapF), cauda epididymal fluid (CauF), and seminal plasma (SemP). **(A)** Immunoblot of total fluid protein in each group probed by PRAMEY antibody and then reprobed by α-tubulin (TUBA) antibody. **(B)** Quantification of pixel intensities for the 58, 30, 26, and 13 kDa PRAMEY bands, normalized to the corresponding TUBA. Data are expressed as the mean of the protein pixel intensity ±SEM for testis (*n* = 5), epididymal fluids (*n* = 5), and seminal plasma (*n* = 9). Significance was evaluated between the four types of fluid (testis, caput, cauda, and seminal plasma) for each PRAMEY isoform (58, 30, 26, and 13 kDa). Values without a common superscript differed (*p* < 0.05).

Evaluation of the fluid collected from the testis, caput, and cauda segment of the epididymis and ejaculate (seminal plasma) revealed a different PRAMEY pattern from that observed for spermatozoa. As shown in Figure 2A, a weak expression of the 58 kDa isoform was observed in all fluid types (Table 1). Where the 58 kDa expression was similar among the fluids from testis and epididymis, its expression level was lower (*p* < 0.05) in seminal plasma (Figure 2A,B). The 30 kDa isoform was strongly detected compared to the 58 kDa and its expression was higher in fluid from testis and caput compared to seminal plasma (*p* < 0.05). Interestingly, the 26 kDa isoform was not observed in fluid from the testis, while it was hardly observed in caput epididymal fluid or seminal plasma, but was mainly observed in fluid from the cauda segment of the epididymis (*p* < 0.05). The 13 kDa isoform was weakly detected in fluid from the caput segment of the epididymis but had a higher (*p* < 0.05) protein concentration observed in fluid from the cauda epididymis and seminal plasma (Figure 2A,B) (Table 1).

### Expression of PRAMEY in the Testicular and Epididymal Tissues

**FIGURE 3:**
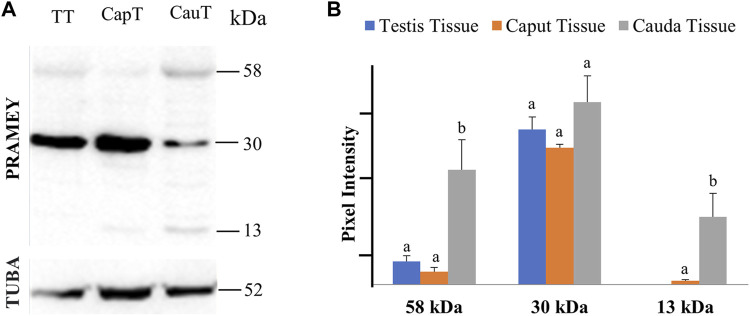
Expression of PRAMEY isoforms in bovine testis tissue (TT) and caput (CapT) and cauda (CauT) epididymal tissues. **(A)** Immunoblot of total tissue protein in each group probed by PRAMEY antibody and then reprobed by α-tubulin (TUBA) antibody. **(B)** Quantification of pixel intensities for the 58, 30, and 13 kDa PRAMEY bands, normalized to the corresponding TUBA. Data are expressed as the mean of the protein pixel intensity ±SEM (*n* = 5). Significance was evaluated between the three types of tissue (testis, caput, cauda) for each PRAMEY isoform (58, 30, and 13 kDa). Values without a common superscript differed (*p* < 0.05).

The presence of PRAMEY in the fluids suggested that PRAMEY may have originated from the testicular and/or epididymal tissue. Therefore, we next evaluated the PRAMEY protein isoform expression in testicular and epididymal tissue (Figure 3A,B). This evaluation showed that the 58 kDa isoform was relatively weakly expressed in testicular and epididymal tissue. However, its expression level was higher in tissue from the cauda segment of the epididymis compared to tissue from the testis and the caput segment of the epididymis (*p* < 0.05) (Figure 3A,B). In contrast to the 58 kDa isoform, the 30 kDa isoform was heavily expressed in all three tissues studied (Table 1), however, no significant difference was observed among them (*p* > 0.05) (Figure 3A,B). Interestingly, the 26 kDa isoform was not observed in the testis or epididymal tissue (Figure 3A,B). The 13 kDa protein expression was weakly detected in caput tissue, while its expression level was higher in cauda tissue (*p* < 0.05) (Figure 3A,B). Moreover, the 13 kDa isoform was not observed in testicular tissue (Figure 3A,B) (Table 1).

### mRNA Expression of PRAMEY in the Testicular and Epididymal Tissues

**FIGURE 4:**
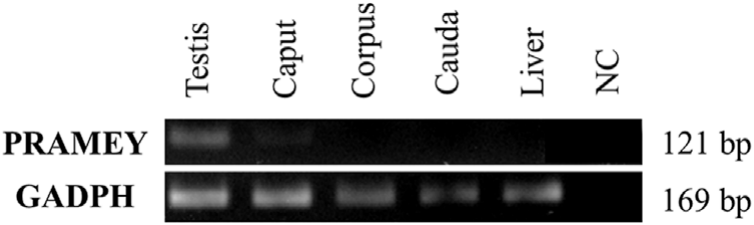
Expression analysis of the bovine *PRAMEY* gene by RT-PCR in bovine testis and epididymal tissues (caput, corpus, and cauda). The liver RNA sample and water (no RNA) were used as negative controls (NC), while the expression of the *GADPH* gene was used as a positive control. PRAMEY was expressed specifically in the testis, while no expression was observed in epididymal and liver tissues.

To address the question of whether the *PRAMEY* gene is expressed in the epididymal tissue, RT-PCR was performed with a pair of *PRAMEY* gene-specific primers on testicular and epididymal tissues (Figure 4). We found that PRAMEY was expressed only in the testis, while no expression was detected in the caput, corpus, or cauda segments of the epididymis, and the negative control, i.e. the liver tissue (Figure 4), confirming our previous report that PRAMEY is germ cell-specific (Liu et al., 2017).

### PRAMEYc Expression Pattern in Spermatozoa, Fluids, and Tissue From Testis and Epididymis

**FIGURE 5:**
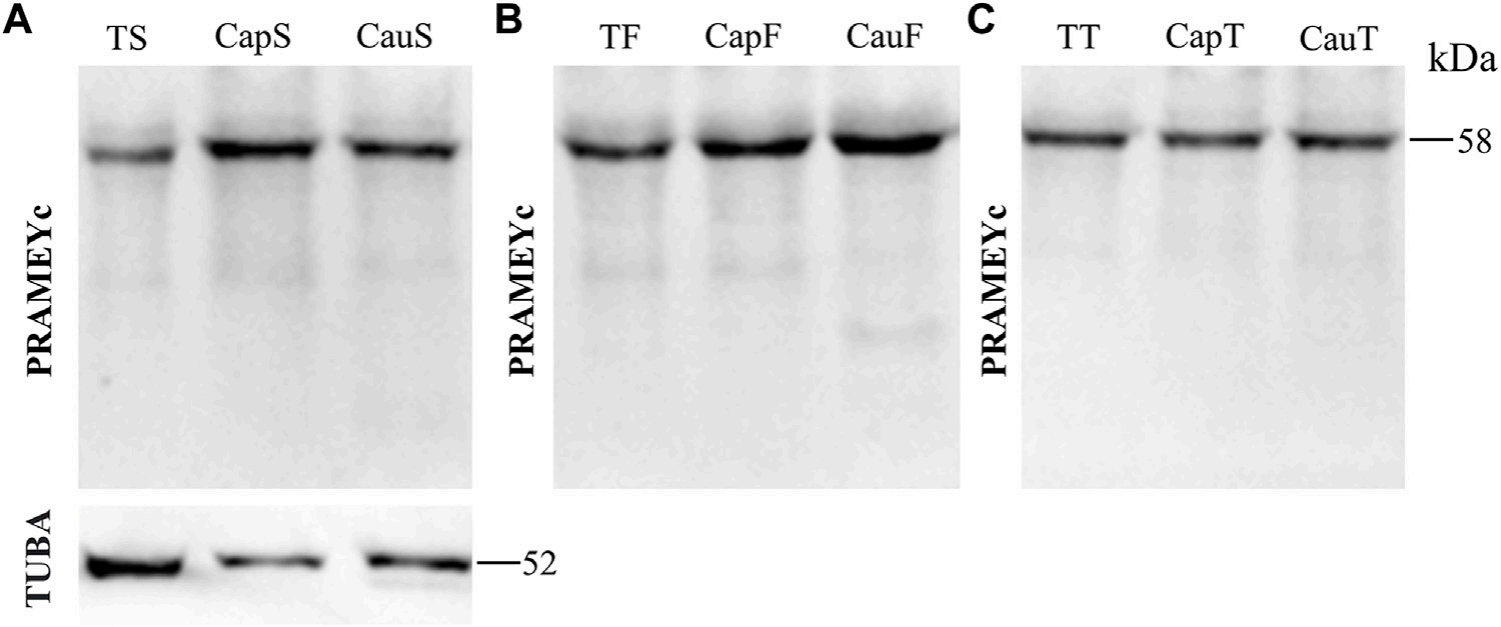
Expression of PRAMEYc in sperm, fluid, and tissue collected from the bovine testis and caput and cauda segments of the epididymis. **(A)** Immunoblot of total testis sperm (TS), caput sperm (CapS), and cauda sperm (CauS) protein probed by the PRAMEYc antibody, then reprobed by α-tubulin (TUBA) antibody. **(B)** Immunoblot of total testis fluid (TF), caput fluid (CapF), and cauda fluid (CauF) protein probed for PRAMEYc content. **(C)** Immunoblot of total testis tissue (TT), caput tissue (CapT), and cauda tissue (CauT) protein probed for PRAMEYc content.

The PRAMEY antibody used in the above experiments was referred to as a N-terminus antibody, which produced four isoforms in the WB results. To determine whether a different antibody designed from the C-terminus of PRAMEY would detect the same isoforms, a custom-made C-terminal PRAMEY antibody (called PRAMEYc) was produced and used in this study. We found that the new PRAMEYc antibody detected the intact 58 kDa PRAMEY only, and the expression level of 58 kDa isoforms was low, but consistent, and no significant variations were found among spermatozoa (Figure 5A), fluid (Figure 5B), and tissue (Figure 5C) from testis and caput and cauda segments of the epididymis (*p* > 0.05). The finding indicated that the 30, 26, and 13 kDa PRAMEY isoforms were generated from the N-terminus of PRAMEY.

### PRAMEY Expression Pattern in Sperm Head and Tail

**FIGURE 6:**
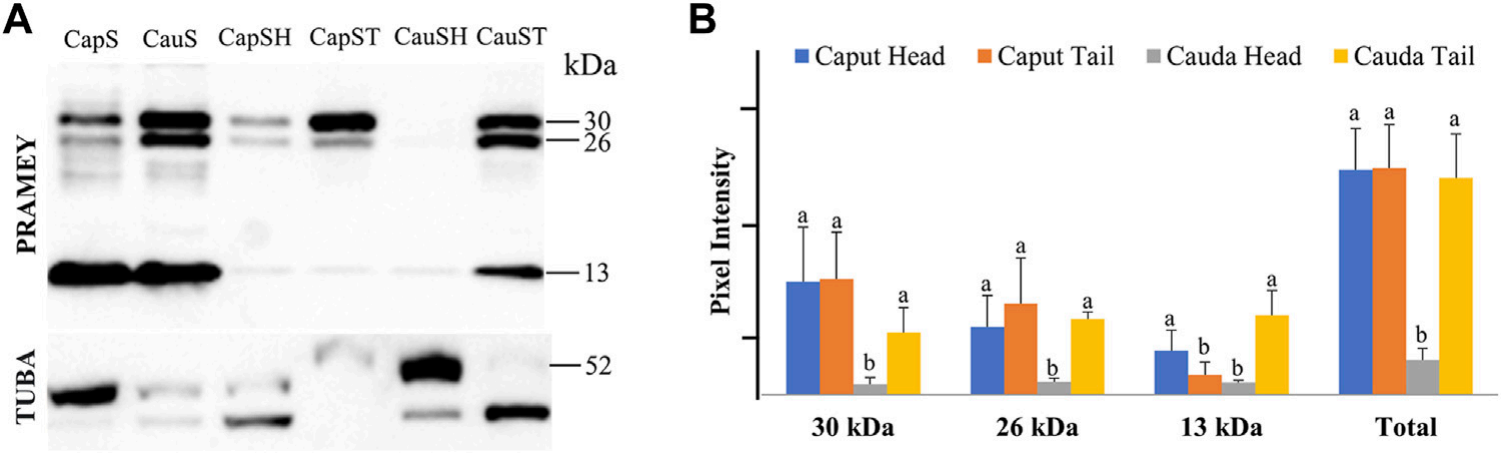
Expression of PRAMEY isoforms in bovine whole caput sperm (CapS), whole cauda sperm (CauS), caput sperm head (CapSH), caput sperm tail (CapST), cauda sperm head (CauSH), and cauda sperm tail (CauST) collected from caput and cauda segments of the epididymis. **(A)** Immunoblot of total sperm protein in each group probed for PRAMEY content and then reprobed for α-tubulin (TUBA). **(B)** Quantification of pixel intensities for the 30, 26, and 13 kDa PRAMEY bands, normalized to the corresponding TUBA. Data are expressed as the mean of the protein density intensity ±SEM (*n* = 3). Significance was evaluated between the four types of sperm (caput head, caput tail, cauda head, and cauda tail) for each PRAMEY isoform (30, 26, and 13 kDa). A different letter indicates a significant difference (*p* < 0.05).

We next evaluated the dynamic of PRAMEY expression between sperm head and tail during maturation in the epididymis (Figure 6A). Among the four PRAMEY isoforms, the 58 kDa isoform was very weakly detected in the separated sperm head and tail, thus it was not evaluated in this experiment. When comparing the 30, 26, and 13 kDa isoforms individually (Figures 6A,B), we found a remarkable decrease of 10.9, 5.4, and 3.8-fold respectively, in protein expression from the caput to cauda epididymis in sperm heads, but there was a small decrease in the expression for both the 30 and 26 kDa isoforms in sperm tails (1.9 and 1.2-fold respectively). In contrast, the 13 kDa isoform increased 4-fold in sperm tails from caput to cauda (Figures 6A,B), suggesting this isoform may have a significant role in tail function, but is likely not as important in sperm head function of cauda spermatozoa. Variations in protein expression were observed among bulls, particularly in the sperm head. For example, the 30 and 26 kDa isoforms in the sperm head of this animal shown in Figure 6A were not detected. When expression levels of all isoforms were combined, PRAMEY expression was nearly equivalent for both caput sperm head and tail (Figure 6B). Alternatively, cauda sperm tails had an expression 6-fold higher than cauda sperm heads, suggesting the role of PRAMEY in tail function (Figure 6B). It is worth noting that α-tubulin antibodies detected two bands from the sperm head and tail samples (Figure 6A). This phenomenon also occurred in α-tubulin detected in caput fluid (Figure 2A).

### Expression of PRAMEY in Subcellular Compartments of Spermatozoa

**FIGURE 7:**
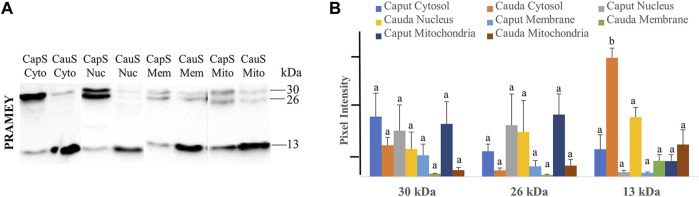
Expression of PRAMEY isoforms in the following subcellular compartments: caput sperm cytosol (CapS Cyto), cauda sperm cytosol (CauS Cyto), caput sperm nucleus (CapS Nuc), cauda sperm nucleus (CauS Nuc), caput sperm membrane (CapS Mem), cauda sperm membrane (CauS Mem), caput sperm mitochondria (CapS Mito), and cauda sperm mitochondria (CauS Mito). **(A)** Immunoblot of total subcellular compartment protein in each group probed by PRAMEY antibody. **(B)** Quantification of pixel intensities for the 30, 26, and 13 kDa PRAMEY bands. Data are expressed as the mean of the protein pixel intensity ±SEM (*n* = 3). Significance was evaluated between the eight types of subcellular compartments (cytosol, nucleus, membrane, and mitochondria from caput and cauda sperm) for each PRAMEY isoform (30, 26, and 13 kDa). Values without a common superscript differed (*p* < 0.05).

To better understand the PRAMEY functional dynamic between head and tail during sperm maturation, we further evaluated the PRAMEY expression in sperm cytosol, nucleus, membrane, and mitochondria during spermatozoal maturation in the epididymis (Figure 7A). Similar to the head and tail experiment, the 58 kDa isoform was very weakly detected, thus it was not evaluated in this experiment. During bovine sperm maturation in the epididymis, the 30 and 26 kDa isoforms tended to decrease in sperm cytosol, nucleus, plasma membrane, and mitochondria from caput to cauda. However, the 13 kDa isoform significantly increased from caput to cauda sperm cytosol (*p* < 0.05), while the nucleus, membrane, and mitochondria expression tended to increase from caput to cauda spermatozoa (Figure 7A,B). The dynamic of PRAMEY localization during sperm transit through epididymis possibly suggests the involvement of PRAMEY in sperm maturation, especially the 13 kDa isoform.

## Discussion

Similar to many other CTA genes/families, the function of the *PRAME* gene family is difficult to dissect due to it being an expanded gene family in the eutherian mammals (Chang et al., 2011). One of the biggest challenges when working with a multi-copy gene/protein family is to design a gene/protein-specific antibody. To tackle this challenge, we have made two PRAMEY-specific antibodies using a PRAMEY-specific short peptide either from the N-terminus (referred to as anti-PRAMEY antibody) (Liu et al., 2017) or from the C-terminus (referred to as anti-PRAMEYc antibody), which were used to examine the PRAMEY isoforms during sperm maturation in this study. Among the four PRAMEY isoforms identified by WB, 58, and 30 kDa were detected in testicular spermatozoa, while the 30, 26, and 13 kDa were found in epididymal spermatozoa. This finding raised a question on whether the different isoforms of PRAMEY were encoded by a different variant of PRAMEY paralogs as the copy number of PRAMEY varies on the Y chromosome from 2 to 30 among healthy bulls (Yue et al., 2013) or were the different isoforms cleavage products from the intact (58 kDa) and 30 kDa protein. One of the best ways to address this question is to sequence the PRAMEY isoforms using an immunoprecipitation-mass spectrometry (IP-MS) approach. Unfortunately, we failed to sequence the PRAMEY proteins after several trials of IP-MS analysis. We noticed that other proteomic studies, particularly the one performed on the isolated mouse chromatoid body (CB), could not identify the PRAME-related proteins either (Meikar et al., 2014). Like bovine PRAMEY, mouse PRAMEX1 (X-linked; previously known as PRAME) and PRAMEL1 (on Chr 4) are highly enriched in the mouse CB (Liu et al., 2021). Failure to identify the bovine and mouse PRAME-related proteins in testis/sperm samples by MS may reveal a challenge in sequencing proteins in the PRAME family.

Although the bovine Y chromosome sequence assembly and gene annotation have not been completely finished yet (Chang et al., 2013), the likelihood of post-testicular translation giving rise to the 26 and 13 kDa from transcript variants of PRAMEY or even from different paralogs of PRAMEY is very low for two possible reasons. One is that there is no evidence to date that the bovine Y chromosome has a copy of *PRAMEY* that encodes a 26 or 13 kDa protein. The other reason is that chromatin of germ cells is undergoing condensation, transcriptional activity is shut down during spermiogenesis, and most of the cytoplasm is lost during sperm maturation (Cooper, 2011; Li et al., 2021; Martins et al., 2021). Therefore, we hypothesize that the 26 and 13 kDa PRAMEY isoforms were a result of cleavage and were released from the spermatozoa into the fluid at some point during spermatozoa maturation.

It was unexpected to find that the 30 kDa PRAMEY protein was heavily present in the fluid of the testis and epididymis, and seminal plasma. The presence of PRAMEY in these fluid samples may be explained by the enrichment of PRAMEY in late the CB and the mitochondria-associated granule (MAG) within the cytoplasm of elongated spermatids (Liu et al., 2017). When the cytoplasm of elongated spermatids is shed at the end of spermiogenesis, the CB, and MAG, which contain PRAMEY, will also be lost from the spermatids through the cytoplasmic droplets. It is believed that the cytoplasmic droplets are released mainly in the caput segment of the epididymis during sperm transition from the testis to cauda epididymis in sperm maturation (Branton and Salisbury, 1947; Cooper and Yeung, 2003; Zhou et al., 2018; Sellem et al., 2021). Therefore, PRAMEY may be shed into epididymal lumen fluid during maturational passage of spermatozoa. Accordingly, the content of 26 and 13 kDa isoforms increases in both epididymal spermatozoa and fluid from caput to cauda. These findings may suggest a transcytosis pathway, where PRAMEY is being transported transcellularly after being released from the spermatid/spermatozoa cytoplasm. If this hypothesis is correct, the PRAMEY protein observed in epididymal fluid and tissue likely originated from spermatozoa (*i.e.* germ cells) during sperm maturation, not from non-germ cells, such as the epididymis. Our RT-PCR results with PRAMEY-specific primers indeed confirmed that the *PRAMEY* gene is expressed in the testis, but not in the epididymal and liver tissues.

Our data from the subcellular compartments of spermatozoa revealed that the 13 kDa PRAMEY isoform increased 4-fold in sperm tails from caput to cauda, suggesting that this isoform may have a significant role in tail function. It is further supported by the data showing that the 13 kDa PRAMEY also increased within mitochondria from caput to cauda sperm, strongly indicating this isoform’s role in spermatozoal motility. This is in line with the previous discovery that PRAMEY interacts with PP1γ2 (Liu et al., 2017), a testis/spermatozoa-specific phosphatase that regulates spermatozoal motility (Smith et al., 1996; Huang et al., 2002; Fardilha et al., 2011; Silva et al., 2021).

In conclusion, the 58 and 30 kDa PRAMEY isoforms are the primary isoforms detected in testicular spermatozoa, fluid, and tissue, suggesting their involvement in spermatogenesis (Table 1). The heavily expressed 30 and 13 kDa PRAMEY isoforms in epididymal and ejaculated spermatozoa strongly suggest their involvement in sperm maturation and fertilizing capability (Table 1). Data from this study and our previous work (Liu et al., 2017; Kern, 2020) supports the proposal that PRAMEY’s function is dynamic during spermatogenesis (Liu et al., 2017), sperm maturation (this work), and sperm-acrosomal function and fertilization (Kern, 2020).

## Data Availability

The original contributions presented in the study are included in the article/Supplementary Material, further inquiries can be directed to the corresponding author.
